# Synthesis of a Non-Symmetrical Disorazole C_1_-Analogue and Its Biological Activity

**DOI:** 10.3390/molecules29051123

**Published:** 2024-03-01

**Authors:** Luca Lizzadro, Oliver Spieß, Silke Reinecke, Marc Stadler, Dieter Schinzer

**Affiliations:** 1 Medicinal Chemistry and Chemical Biology Laboratory, School of Pharmacy, University of California San Francisco, 600 16th St., San Francisco, CA 94158, USA; luca.lizzadro@ucsf.edu; 2 Chemisches Institut, Otto-von-Guericke-Universität, Universitätsplatz 2, 39106 Magdeburg, Germany; oliver.spiess@ovgu.de; 3 Helmholtz-Zentrum für Infektionsforschung GmbH, Inhoffenstraße 7, 38124 Braunschweig, Germany; silke.reinecke@helmholtz-hzi.de (S.R.); marc.stadler@helmholtz-hzi.de (M.S.); 4 Institute of Microbiology, Technische Universität Braunschweig, Spielmannstraße 7, 38106 Braunschweig, Germany

**Keywords:** non-symmetrical disorazole, convergent synthesis, biological activity, SAR (structure-activity-relationship)

## Abstract

The synthesis of a novel disorazole C_1_ analogue is described, and its biological activity as a cytotoxic compound is reported. Based on our convergent and flexible route to the disorazole core, we wish to report a robust strategy to synthesize a non-symmetrical disorazole in which we couple one half of the molecule containing the naturally occurring oxazole heterocycle and the second half of the disorazole macrocycle containing a thiazole heterocycle. This resulted in a very unusual non-symmetrical disorazole C_1_ analogue containing two different heterocycles, and its biological activity was studied. This provided exciting information about SAR (structure-activity-relationship) for this highly potent class of antitumor compounds.

## 1. Introduction

**Figure 1:**
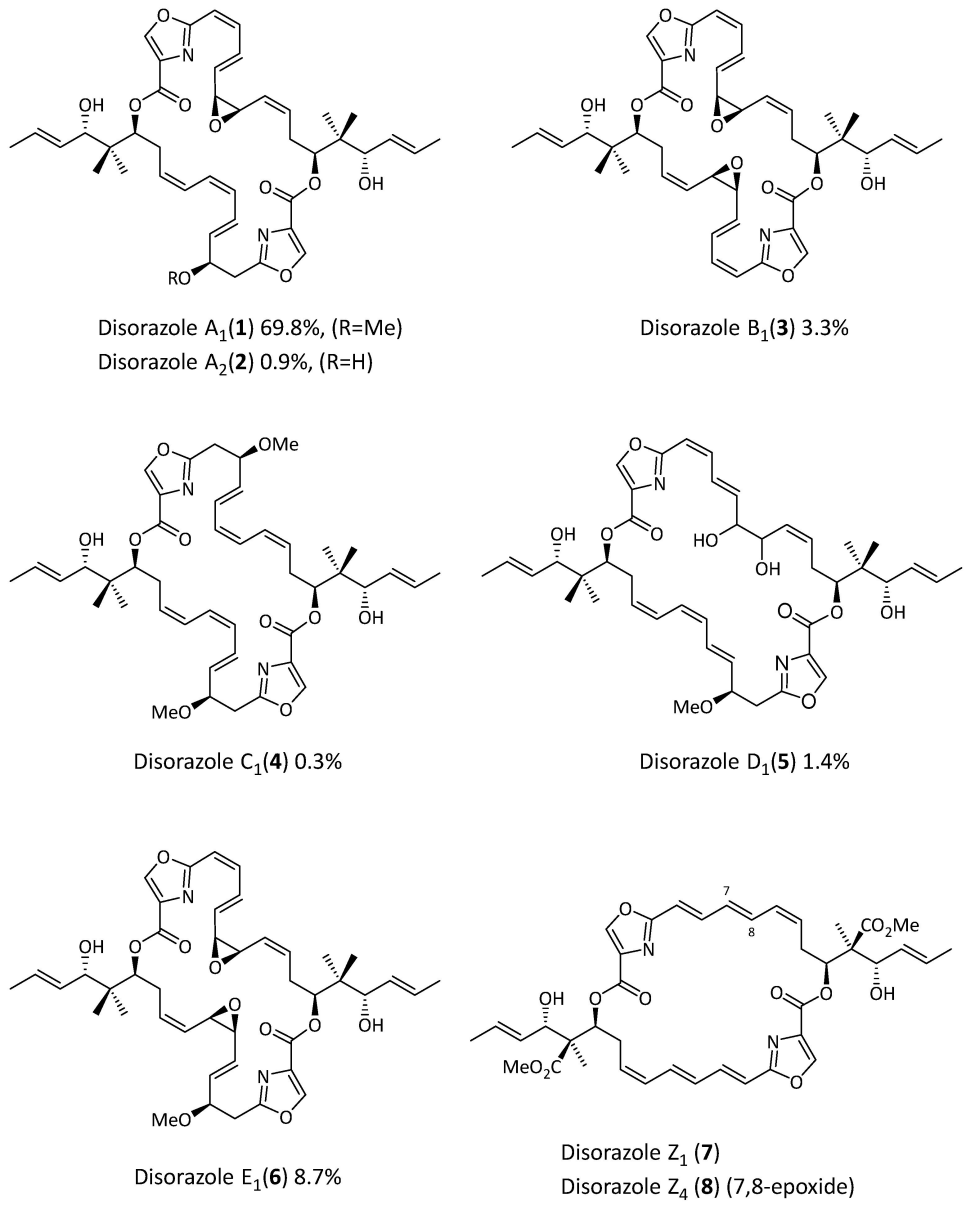
Selected members of the disorazole family. The percentages correspond to the relative abundance from the initial isolation from *Sorangium cellulosum,* So ce12.

The disorazoles are a family of 39 macrolides isolated so far, showing macrolide ring sizes between 26 and 32 (Figure 1) [1,2,3]. They are secondary metabolites from the Myxobacterium *Sorangium cellulosum* (So ce12) and were isolated by the research groups of Höfle and Reichenbach in 1994 [4].

All these natural products show very potent antitumor activity due to inhibition of tubulin polymerization combined with a very powerful cytotoxicity up to picomolar activity against various human cancer cell lines [5,6]. This exciting biological profile generated a tremendous interest in the scientific community in both total synthesis and biology [7]. In addition, their enormous biological potency makes them very attractive in personalized medicine as potesntial payloads for antibody-drug conjugates (ADCs) in targeted cancer therapy [8].

We recently published a flexible and robust new route to synthesize (—)-disorazole C_1_, which involved, at the endgame, a coupling of the building blocks via Yamaguchi esterification and a final Yamaguchi macrolactonization [9].

The advantage of this powerful strategy to construct the disorazole core is that it offers high diversity in each building block before the desired coupling takes place and provides great opportunities to design a variety of disorazole analogues to study SAR (structure-activity relationships) [10].

Based on this strategy, we wish to show our efforts along these lines and report an efficient synthesis to construct a non-symmetrical disorazole C_1_ analogue with potent antitumor activity. Most of the published analogues of this highly active natural product family are based on symmetrical compounds, and to the best of our knowledge, only one non-symmetrical disorazole synthesis has been published in the past by K. C. Nicolaou [11].

## 2. Results

In a recent study, we focused on the structure-activity relationship of the disorazole C_1_ core by exchanging the oxazole ring with thiazole, and we studied the influence of chiral centers within the disorazole framework [10]. Employing the same strategy we successfully used in our total synthesis of disorazole C_1_, we have prepared an analogue with the oxazole ring replaced by a thiazole unit. To our surprise, this replacement resulted in an increase in IC_50_ values between 200- and 800-fold. Even more dramatic was the inversion of configuration at C(14)/C(14’) stereocenters, with a drop in activity of several thousand-fold [10].

Much less influence had the inversion of configuration at C(5)/C(5’) stereocenters, with a loss of activity of only between 40- and 160-fold [10].

Based on these findings, it would be of great interest to synthesize a kind of hybrid structure connecting one half of the C2-symmetric macrocycle containing the natural oxazole heterocycle and the other half containing a thiazole unit.

Our published strategy offers the possibility of synthesizing a large variety of disorazole analogues. In order to construct hybrid **16,** one just has to construct the two halves **9** and **10**. Fragment **10** can be used from the total synthesis of disorazole C_1_, and the thiazole fragment **9** can be used from the synthesis of bis-thiazole **17**.

**Scheme 1:**
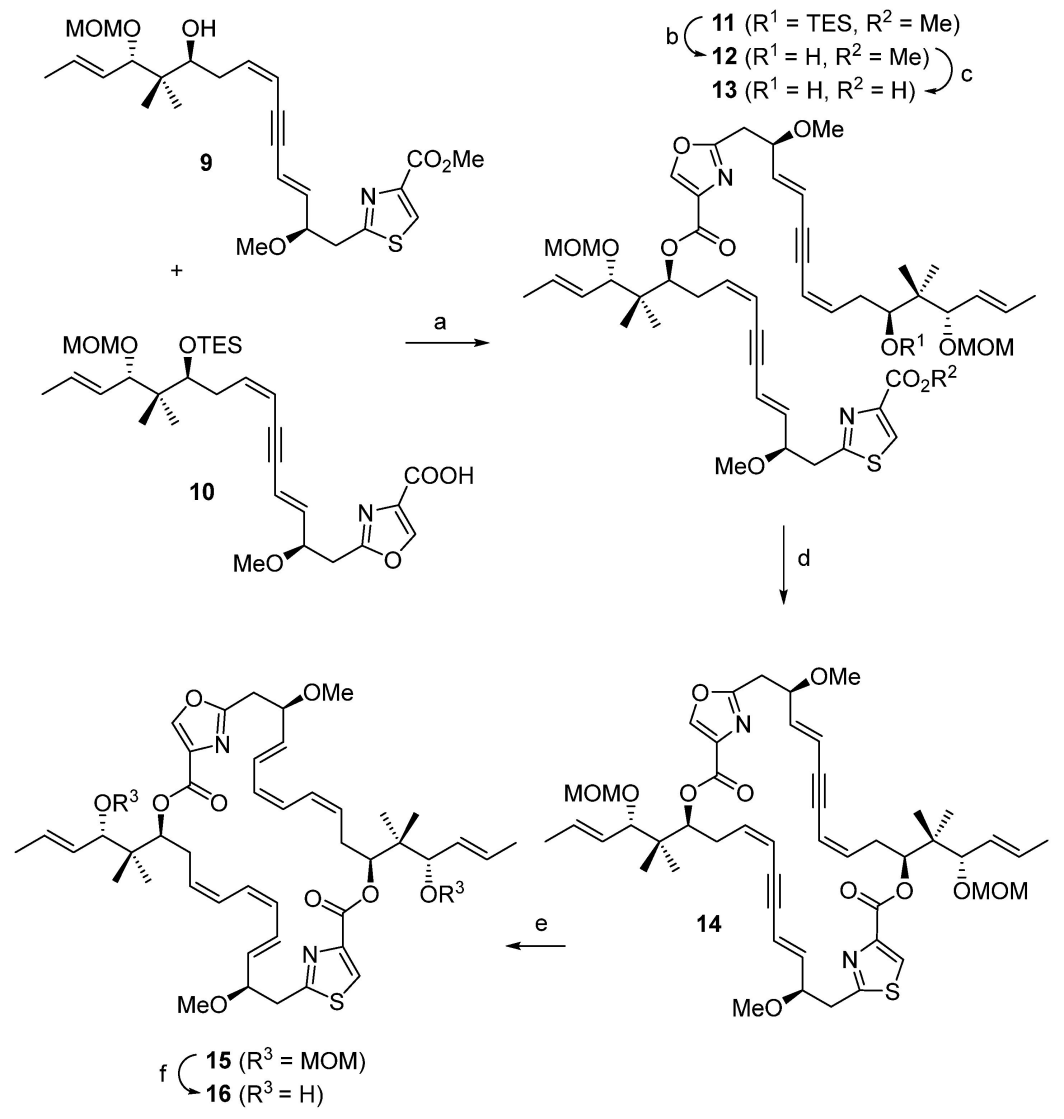
Reagents and conditions: (a) 2,4,6-trichlorobenzoyl chloride, NEt_3_, DMAP, THF/toluene, rt, 18 h, 81%; (b) CSA, DCM/MeOH, 0 °C, 1 h; (c) LiOH, THF/H_2_O, rt, 16 h; (d) 2,4,6-trichlorobenzoyl chloride, NEt_3_, DMAP, THF/toluene, rt 18 h, 58% from **10**; (e) activated zinc, MeOH/H_2_O, 50 °C, 24 h, 62%; (f) HBr, ACN, −15 °C, 2 h 30 min, 50%. NEt_3_ = triethylamine; DMAP = 4-dimethylaminopyridine; CSA = camphorsulfonic acid; LiOH = lithium hydroxide; HBr = hydrogen bromide; ACN = acetonitrile.

Alcohol **9** was esterified using the Yamaguchi protocol with acid **10** to obtain compound **11** at 81% as a single compound (Scheme 1). Mild deprotection of TES-protected compound **11** with CSA provided the desired alcohol **12,** which was directly hydrolyzed with LiOH, providing the starting material **13** for the Yamaguchi macrolactonization. Macrocycle **14** was isolated in 58% yield as a single isomer, which was transferred to the required (*E*,*Z*,*Z*)-triene derivative **15** in 62% using the Boland hydrogenation protocol.

Very simple deprotection of the bis-MOM compound **15** with two drops of HBr at −15 °C in CH_3_CN provided the disorazole analogue **16** in 50% yield.

**Figure 2:**
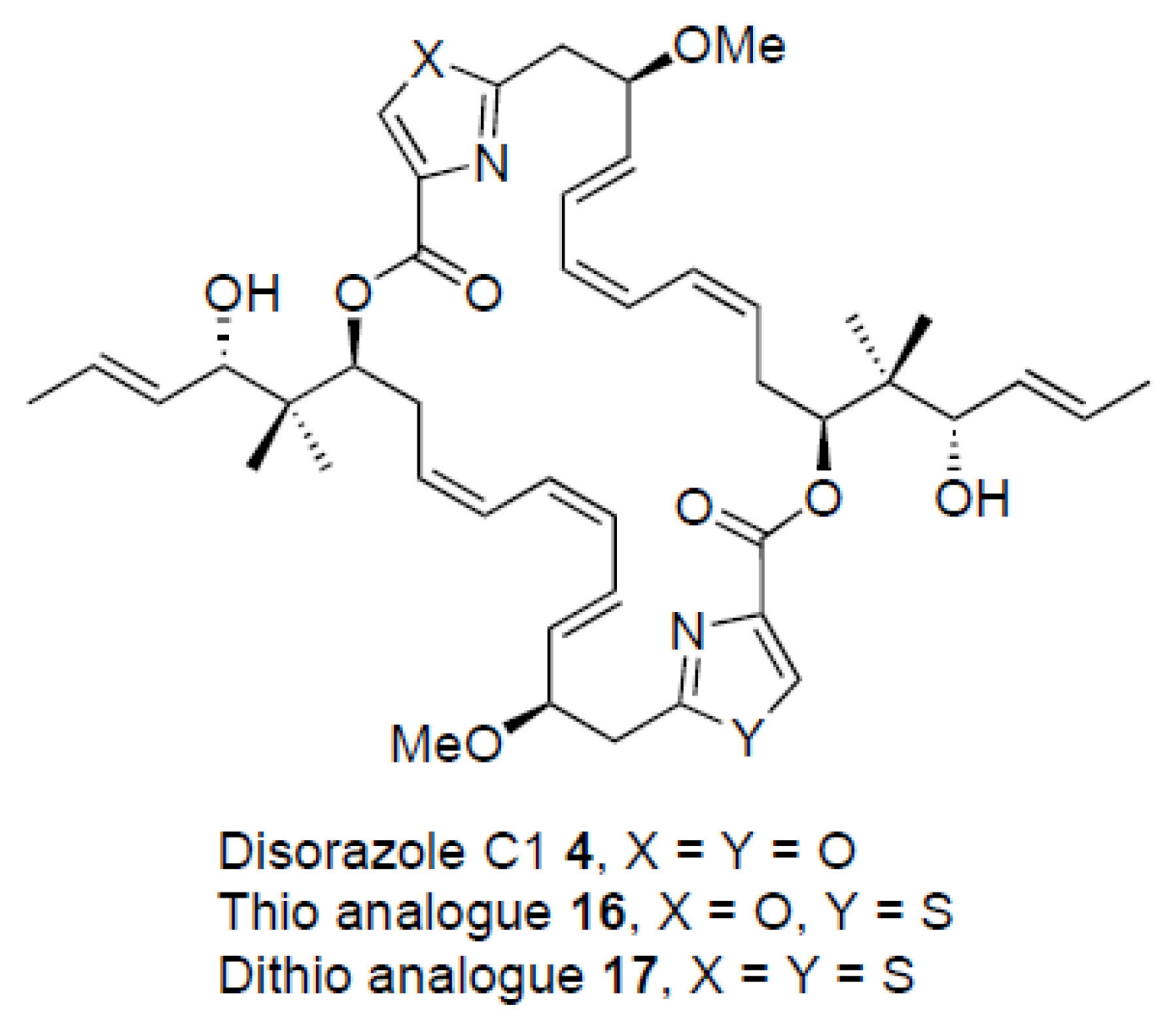
Disorazole C_1_ and analogues tested.

After completion of the synthesis of **16,** the biological activity of the analogue was evaluated against several immortalized animal and human cancer cell lines and compared to the cytotoxicity of synthetic disorazole C_1_ and epothilone B as internal controls (Figure 2) [12,13].

Table 1 shows these results and demonstrates again the very high activity of disorazole C_1_ in the low sub-nanomolar range (between 0.11 and 0.6 ng/mL). Compound **16** is only one order of magnitude less active than the natural product **4**, but several orders of magnitude more active than the bis-thiazole analogue **17**.

## 3. Discussion

The hybrid disorazole **16** provides a very important piece of information to understand the biological activity of disorazoles because we can compare a series of analogues and can now offer a rationale to understand the activity differences and bring some light into the structure-activity relationship (SAR) of these fascinating molecules.

So far, only for disorazole Z, an X-ray exists to provide competitive data about the interaction of the molecule with the target protein tubulin [14,15]. Given the fact that more or less the same interactions take place with other disorazoles, only one half of disorazole C_1_ is involved in protein binding to tubulin, and therefore, this can explain the activity differences with our compounds.

Disorazole C_1_ is still the most active compound in our series, whereas bis-thiazole **17** showed around 200–800 fold less activity. However, hybrid **16** still retained most of the activity of the natural product **4,** with an increased IC_50_ value of about a maximum of 10. This clearly demonstrates that the natural oxazole half of **16** preferentially binds to the protein, leaving the thiazole half outside of the close interaction with tubulin. In addition, it explains the quite large drop in activity of the bis-thiazole compound **17**.

## 4. Materials and Methods

Solvents were dried by standard procedures and redistilled under an N_2_ atmosphere prior to use. All reactions were run under nitrogen, unless otherwise stated. For reactions that require heating, an oil bath was used as an external heat source. When the reactions were run at room temperature, a temperature of 22 °C ± 2 °C was implied. The products were purified by flash chromatography on Merck silica gel 60 (40–63 μm). POLYGRAM SIL G/UV254 prefabricated TLC plates with fluorescent indicators from Macherey-Nagel have been used for analytical thin layer chromatography (TLC). The separated substances were detected by irradiation with UV light with a wavelength of 254 nm, staining with vanillin or potassium permanganate reagent, and subsequent warming with a heat gun. Electrospray ionization (ESI) mass spectra were recorded on Waters Xevo G2-TOF spectrometers. ^1^H and ^13^C-NMR spectra were recorded on Bruker AVIII 400 and Bruker AVI 600 spectrometers (Supplementary Materials). Chemical shifts (δ) are reported in ppm, referencing the resonance signal of the residual undeuterated solvent for ^1^H- NMR and the deuterated solvent for ^13^C-NMR (^1^H-NMR = CDCl_3_: δ 7.26, CD_3_OD: δ 3.31; ^13^C-NMR = CDCl_3_: δ 77.16, CD_3_OD: δ 49.00). Data are reported as follows: chemical shift, multiplicity (s = singlet, d = doublet, t = triplet, br = broad, m = multiplet, app = apparent), coupling constants (Hz), and integration. Optical rotations were recorded on Perkin-Elmer 341 and Anton Paar MCP150 polarimeters employing the solvent and concentration indicated and are reported in units of 10^−1^ (deg cm^2^ g^−1^). 

**11**: NEt_3_ (90 µL, 0.648 mmol, 6 eq) and 2,4,6-trichlorobenzoyl chloride (68 µL, 0.432 mmol, 4 eq) were added to a solution of carboxylic acid **10** (91 mg, 0.162 mmol, 1.5 eq) in THF (5 mL), and the resulting turbid solution was stirred for 2 h at room temperature. Toluene (3 mL) was added, and then the mixture was added dropwise to a solution of alcohol **9** (50 mg, 0.108 mmol) and DMAP (79 mg, 0.648 mmol, 6 eq) in toluene (5 mL). The mixture was stirred for 16 h at room temperature and then quenched with a saturated aqueous NH_4_Cl solution (15 mL). The aqueous phase was extracted with EtOAc (3 × 10 mL). The organic layers were dried over Na_2_SO_4_, filtered, and concentrated in vacuo. The residue was purified by flash chromatography (Et_2_O/pentane 2:1) to afford **11** (83 mg, 0.0813 mmol, 81%) as a slightly yellow oil. **General Data**: C_55_H_80_N_2_O_12_SSi; MW: 1021.39; TLC: Rf = 0.30 (pentane/Et_2_O 2:1); UV (+); Vanillin: black; ${\left[\alpha \right]}_{D}^{20}$ = +24.44 (*c* = 0.9, CHCl_3_). 

**^1^H-NMR** (400 MHz, CD_3_OD): δ (ppm): 8.47 (s, 1H); 8.25 (s, 1H); 6.10–6.23 (m, 1H); 6.02–5.94 (m, 4H); 5.90–5.80 (m, 1H); 5.72–5.58 (m, 4H); 5.47–5.33 (m, 2H); 5.25 (app t, *J* = 6.6 Hz, 1H); 4.69–4.59 (m, 2H); 4.50–4.37 (m, 2H); 4.24–4.16 (m, 1H); 3.88 (s, 3H); 3.89–3.83 (m, 2H); 3.34 (s, 3H); 3.33 (s, 3H); 3.28 (s, 3H); 3.27 (s, 3H); 3.44–2.98 (m, 3H); 2.81–2.70 (m, 2H); 2.65–2.54 (m, 2H); 2.48–2.39 (m, 1H); 1.72 (ddd, *J* = 12.8, 6.6, 1.6 Hz, 6H); 0.984 (app t, *J* = 7.6 Hz, 9H); 1.01 (s, 3H); 0.96 (s, 3H); 0.94 (s, 3H); 0.87 (s, 3H); 0.64 (q, *J* = 7.7 Hz, 6H). **^13^C NMR** (101 MHz, CD_3_OD): δ (ppm): 164.41, 164.38, 162.87, 162.22, 146.08, 143.59, 142.00, 141.81, 141.66, 136.61, 133.61, 133.16, 132.34, 129.19, 128.63, 128.51, 114.76, 114.57, 112.11, 110.81, 94.64, 94.52, 92.57, 88.92, 88.49, 82.75, 81.37, 80.08, 78.59, 77.90, 66.91, 61.53, 57.12, 57.07, 56.33, 55.97, 52.48, 44.22, 42.85, 35.50, 35.04, 34.65, 32.40, 19.73, 19.54, 18.05, 18.03, 15.45, 14.47, 7.52, 6.52. **HRMS** (ESI) *m*/*z*: [*M*+H]^+^ Calcd for C_55_H_81_N_2_O_12_SSi: 1021.5279; found: 1021.5275.

**14**: TES-protected alcohol **11** (80.3 mg, 0.0787 mmol) was dissolved in CH_2_Cl_2_/MeOH 1:1 (4 mL, 0.02 M) and cooled to 0 °C. CSA (3.7 mg, 0.0157 mmol, 0.2 eq) was added, and the mixture was stirred for 1 h at 0 °C under a normal atmosphere. Saturated aqueous NaHCO_3_ solution (5 mL) was added, and the layers were separated. The aqueous phase was extracted with CH_2_Cl_2_ (3 × 5 mL), and the combined organic extracts were dried over Na_2_SO_4_, filtered, and concentrated in vacuo. The crude material was used in the next step without further purification. **General Data**: C_49_H_66_N_2_O_12_S; MW: 907.13; TLC: Rf = 0.30 (CH_2_Cl_2_/MeOH 50:1); UV (+); Vanillin: black; ${\left[\alpha \right]}_{D}^{20}$ = +40.4 (*c* = 0.5, CHCl_3_). **HRMS** (ESI) *m*/*z*: [*M*+H]^+^ Calcd for C_49_H_67_N_2_O_12_S: 907.4415; found: 907.4415. Deprotected alcohol **12** (71.3 mg, 0.0787 mmol) was dissolved in THF (2 mL) and LiOH (1 M in H_2_O, 0.236 mL, 0.236 mmol, 3 eq) was added. The mixture was stirred at room temperature for 16 h and neutralized with 1 M HCl (~1 mL). The aqueous phase was extracted with Et_2_O (3 × 3 mL), and the organic extracts were dried over Na_2_SO_4_, filtered, and concentrated in vacuo to give crude *seco*-acid **13**, which was used for the next step without further purification. **General Data**: C_48_H_64_N_2_O_12_S; MW: 893.10; TLC: UV (+); Vanillin: black; ${\left[\alpha \right]}_{D}^{20}$ = +24.4 (*c* = 0.5, CHCl_3_). **HRMS** (ESI) *m*/*z*: [*M*+H]^+^ Calcd for C_48_H_65_N_2_O_12_S: 893.4258; found: 893.4261. NEt_3_ (219 μL, 1.57 mmol, 20 eq) and 2,4,6-trichlorobenzoyl chloride (123 μL, 0.787 mmol, 10 eq) were added at room temperature to a solution of crude **13** (66 mg, 0.0787 mmol) in THF (5 mL), and this turbid solution was stirred for 2 h at room temperature. Toluene (2 mL) was added, and the solution was added dropwise to a solution of DMAP (385 mg, 3.15 mmol, 40 eq) in toluene (40 mL). The mixture was stirred overnight at room temperature, and then quenched with saturated aqueous NH_4_Cl solution (10 mL), water (10 mL), and the aqueous phase was extracted with EtOAc (3 × 20 mL). The organic layers were dried over Na_2_SO_4_, filtered, and concentrated in vacuo. The residue was purified by flash chromatography (hexane/EtOAc 2:1 to 1:1) to afford the macrocycle **14** (40 mg, 0.0457 mmol, 58% from **11**) as a yellow wax. **General Data**: C_48_H_62_N_2_O_11_S; MW: 875.09; TLC: Rf = 0.50 (CH_2_Cl_2_/MeOH 50:1); UV (+); Vanillin: black; ${\left[\alpha \right]}_{D}^{20}$ = +160.40 (*c* = 0.5, CHCl_3_). **^1^H-NMR** (400 MHz, CDCl_3_): δ (ppm): 8.05 (s, 1H); 7.98 (s, 1H); 6.03–5.89 (m, 4H); 5.84–5.71 (m, 2H); 5.70–5.57 (m, 3H); 5.52 (m, 1H); 5.46–5.27 (m, 4H); 4.65 (dd, *J* = 16.0, 6.5 Hz, 2H); 4.40 (dd, *J* = 16.2, 6.7 Hz, 2H); 4.17–4.07 (m, 2H); 3.75 (dt, *J* = 9.0, 3.4 Hz, 2H); 3.38 (s, 3H); 3.36 (s, 3H); 3.35 (s, 3H); 3.34 (s, 3H); 3.29 (m, 2H); 3.26–3.14 (m, 2H); 3.07–2.94 (m, 2H); 2.47–2.37 (m, 2H); 1.72 (m, 6H); 1.07 (s, 3H); 1.05 (s, 3H); 1.03 (s, 3H); 1.00 (s, 3H). **^13^C NMR** (101 MHz, CDCl_3_): δ (ppm): 165.71, 161.83, 160.63, 160.62, 147.20, 147.27, 143.55, 140.76, 140.70, 140.33, 133.77, 132.01, 131.89, 127.39, 127.28, 114.51, 113.42, 112.21, 112.11, 93.83, 93.78, 91.08, 91.02, 87.98, 87.96, 81.64, 81.48, 80.35, 79.39, 77.36, 76.67, 57.07, 56.93, 56.19, 56.17, 41.69, 41.64, 39.07, 34.51, 31.73, 31.60, 20.04, 19.86, 19.69, 19.64, 18.10, 18.07. **HRMS** (ESI) *m*/*z*: [*M*+H]^+^ Calcd for C_48_H_63_N_2_O_11_S: 875.4330; found: 875.4332.

**15**: Nitrogen was bubbled for 15 min through a suspension of zinc dust (5.00 g, 45.88 mmol) in H_2_O (30 mL), and then Cu(OAc)·2H_2_O (500 mg, 2.5 mmol) was added at room temperature. After 15 min, AgNO_3_ (500 mg, 2.94 mmol) was added carefully. The mixture was stirred for 30 min at room temperature, filtered by suction, and finally washed with H_2_O (40 mL), MeOH (30 mL), acetone (30 mL), and Et_2_O (30 mL). This activated zinc solid was added to a solution of **14** (31.5 mg, 0.0360 mmol) in MeOH/H_2_O 1:1 (20 mL), nitrogen was once again bubbled through the suspension for 10 min, and then the flask was sealed. The mixture was stirred for 24 h at 50 °C, then filtered on a small pad of silica with MeOH washes. The filtrate was concentrated in vacuo and the residue was purified by flash chromatography (CH_2_Cl_2_/MeOH 70:1) to afford **15** (19 mg, 0.0216 mmol, 62%) as a colorless wax. **General Data**: C_48_H_66_N_2_O_11_S; MW: 879.12; TLC: Rf = 0.40 (CH_2_Cl_2_/MeOH 50:1); UV (+); Vanillin: dark green; ${\left[\alpha \right]}_{D}^{20}$ = −19.9 (*c* = 1.0, CHCl_3_). **^1^H-NMR** (600 MHz, CDCl_3_): δ (ppm): 7.89 (s, 1H); 7.82 (s, 1H); 6.47–4.26 (m, 5H); 6.18 (dd, *J* = 19.7, 10.4 Hz, 1H); 6.95–6.83 (m, 2H); 5.67–5.58 (m, 3H); 5.57–5.47 (m, 3H); 5.43–5.36 (m, 2H); 5.31–5.25 (m, 2H); 4.68–4.63 (m, 2H); 4.43–4.36 (m, 2H); 4.16–4.08 (m, 2H); 3.72 (dd, *J* = 18.9, 8.9 Hz, 2H); 3.42–3.36 (m, 2H); 3.33 (s, 3H); 3.32 (s, 3H); 3.28 (s, 3H); 3.27 (s, 3H); 3.19–3.09 (m, 2H); 2.99 (dd, *J* = 14.0, 7.6 Hz, 1H); 2.81 (dd, *J* = 14.7, 8.1 Hz, 1H); 2.50–2.43 (m, 1H); 2.40–2.31 (m, 1H); 1.72 (ddd, *J* = 9.0, 5.3, 4.5 Hz, 6H); 1.03 (s, 3H); 1.01 (s, 3H); 0.98 (s, 3H); 0.94 (s, 3H). **^13^C NMR** (151 MHz, CDCl_3_): δ (ppm): 166.53, 162.41, 160.75, 160.74, 147.20, 143.24, 133.53, 133.22, 132.99, 132.11, 132.05, 130.17, 129.75, 129.61, 129.43, 128.18, 128.10, 127.38, 127.22, 127.14, 126.70, 125.61, 125.52, 125.44, 93.62, 81.87, 81.69, 81.63, 81.11, 80.14, 77.57, 56.67, 56.58, 56.13, 56.11, 41.72, 41.69, 39.74, 35.14, 29.84, 28.39, 20.32, 20.16, 20.00, 19.90, 18.08, 18.06. **HRMS** (ESI) *m*/*z*: [*M*+H]^+^ Calcd for C_48_H_67_N_2_O_11_S: 879.4466; found 879.4461. 

**16**: Compound **15** (17.5 mg, 19.91 μmol) was dissolved in acetonitrile (2 mL) and cooled to −15 °C. HBr (two drops, 48% in H_2_O) was added dropwise, and the mixture was stirred for 2 h 30 min at −15 °C. EtOAc (5 mL) was added, and the mixture was washed with saturated aqueous NaHCO_3_ solution (5 mL). The aqueous phase was extracted with EtOAc (3 × 5 mL), and the organic extracts were dried over Na_2_SO_4_, filtered, and concentrated in vacuo. The residue was purified by flash chromatography (CH_2_Cl_2_/MeOH 50:1) to give thiazolyl-disorazole C_1_ (**16**) (7.9 mg, 9.95 μmol, 50%) as a colorless wax. **General Data**: C_44_H_58_N_2_O_9_S; MW: 791.01; TLC: Rf = 0.20 (CH_2_Cl_2_/MeOH 50:1); UV (+); Vanillin: dark green; ${\left[\alpha \right]}_{D}^{20}$ = −85.9 (C = 0.191 in MeOH). **^1^H-NMR** (600 MHz, CD_3_OD): δ (ppm): 8.26 (s, 1H); 8.21 (s, 1H); 6.54–6.45 (m, 2H); 6.43–6.34 (m, 2H); 6.27 (dd, *J* = 21.4, 10.5 Hz, 2H); 5.93 (app t, *J* = 11.0 Hz, 1H); 5.83 (dd, *J* = 10.7, 5.9 Hz, 1H); 5.71–5.62 (m, 2H); 5.62–5.53 (m, 2H); 5.53–5.42 (m, 4H); 5.26 (app dt, *J* = 14.0, 2.7 Hz, 2H); 4.14 (dd, *J* = 13.7, 7.4 Hz, 2H); 3.86 (dd, *J* = 20.7, 8.1 Hz, 2H); 3.25 (s, 3H); 3.22 (s, 3H); 3.04–2.96 (m, 2H); 2.78 (dd, *J* = 15.5, 5.7 Hz, 2H); 2.72–2.62 (m, 2H); 2.48–2.39 (m, 2H); 1.70 (ddd, *J* = 11.8, 6.4, 1.3 Hz, 6H); 1.03 (s, 3H); 1.01 (s, 3H); 0.98 (s, 3H); 0.95 (s, 3H). **^13^C NMR** (151 MHz, CD_3_OD): δ (ppm): 168.90, 164.27, 162.25, 147.55, 145.76, 134.42, 134.10, 133.80, 131.70, 131.66, 130.85, 130.60, 130.18, 129.88, 129.67, 129.62, 129.43, 129.13, 129.03, 127.32, 127.15, 126.77, 126.55, 81.66, 80.77, 79.13, 78.78, 77.91, 77.83, 56.89, 56.78, 42.81, 42.75, 40.42, 35.96, 29.28, 29.09, 19.46, 19.42, 19.31, 18.04. **HRMS** (ESI) *m*/*z*: [*M*+H]^+^ Calcd for C_44_H_59_N_2_O_9_S: 791.3941; found 791.3938. 

## 5. Conclusions

The disorazoles are a fascinating group of natural products that attracted both the synthetic chemistry community and biologists. Within the last decade, quite a few research groups have reported new total synthesis efforts and strategies to synthesize these attractive molecules. In addition, several SAR studies have been reported to shed some light on the biology of these interesting compounds. Our results provide some more rationale for these findings for the future because still no in vivo data is reported about the therapeutic usefulness of these highly active molecules. Based on the outstanding cytotoxicity of the compounds, it is still very reasonable that they could be powerful payloads for ADCs.

## Figures and Tables

**Table 1:** Biological activities of Disorazole C_1_ and thio analogues. Epothilone B was used as internal standard.

	Cell Line					
	L929	KB3.1	A431	A549	PC-3	MCF-7
Compound	IC_50_ (ng/mL)					
Epothilone B	0.24	0.017	0.026	0.034	0.048	0.015
Disorazole C_1_ **4**	0.25	0.23	0.32	0.60	0,11	0.28
Analogue **16**	2.1	0.94	0.94	1.6	1.7	0.75
Analogue **17**	180	85	82	86	120	91

## Data Availability

Data are contained within the article and Supplementary Materials.

## Supplementary Materials

The following supporting information can be downloaded at: https://www.mdpi.com/article/10.3390/molecules29051123/s1, ^1^H- and ^13^C-NMR spectra of synthesized compounds.

## References

[1] Jahresbericht GBF 2001. https://www.helmholtz-hzi.de/fileadmin/user_upload/Infothek/Ueber_das_HZI/Jahresberichte/Ergebnisberichte/Annual_Report_2001.pdf.

[2] Irschik H., Jansen R., Gerth K., Höfle G., Reichenbach H. (1995). Disorazole A_1_, an Efficient Inhibitor of Eukariotic Organisms Isolated from Myxobacteria. J. Antibiot..

[3] Weissman K.J., Mueller R. (2010). Myxobacterial secondary metabolites: Bioactivities and modes-of-action. Nat. Prod. Rep..

[4] Jansen R., Irschik H., Reichenbach H., Wray V., Höfle G. (1994). Disorazoles, Highly Cytotoxyc Metabolites from the Sorangicin–Producing Bacterium *Sorangium cellulosum*. Liebigs Ann. Chem..

[5] Elnakady Y.A., Sasse F., Lünsdorf H., Reichenbach H. (2004). Disorazol A_1_, a highly effective antimitotic agent acting on tubulin polymerization and inducing apoptosis in mammalian cells. Biochem. Pharmacol..

[6] Gao Y., Birkelbach J., Fu C., Herrmann J., Irschik H., Morgenstern B., Hirschfelder K., Li R., Zhang Y., Jansen R. (2023). The Disorazole Z Family of Highly Potent Anticancer Natural Products from *Sorangium cellulosum*: Bioactivity, Biosynthesis, and Heterologous Expression. Microbiol. Spectr..

[7] Bold C.P., Altmann K.-H. (2024). The Chemistry of Disorazoles and Structure-Activity Relationships: An Update. Tetrahedron.

[8] Perez H.L., Cardaerelli P.M., Deshpande S., Gangwar S., Schroeder G.M., Vite G.D., Borzilleri R.M. (2014). Antibody-drug conjugates: Current status and future directions. Drug Discov. Today.

[9] Lizzadro L., Spieß O., Schinzer D. (2021). Total Synthesis of (−)-Disorazole C_1_. Org. Lett..

[10] Lizzadro L., Spieß O., Collisi W., Stadler L., Schinzer D. (2022). Extending the Struture-Activity Relationship of Disorazole C_1_: Exchanging the Oxazole Ring by Thiazole and Influence of Chiral Centers within Disorazole Core on Cytotoxicity. ChemBioChem.

[11] Nicolaou K.C., Bellavance G., Buchman M., Pulikuri K.K. (2017). Total Synthesis of Disorazoles A_1_ and B_1_ and full Structural Elucidation of Disorazole B_1_. J. Am. Chem. Soc..

[12] Shao L., Marin-Felix Y., Surup F., Stchigel A.M., Stadler M. (2020). Seven New Cytotoxic and Antimicrobial Xanthoquinodins from *Jugulospora vestita*. J. Fungi.

[13] Becker K., Wessel A.-C., Luangsa-ard J.J., Stadler M. (2020). Viridistratins A-C, Antimicrobial and Cytotoxic Benzo[*j*]fluoranthenes from Stromata of *Annulohypoxylon viridistratum* (Hypoxylaceae, Ascomycota). Biomolecules.

[14] Menchon G., Prota A.E., Lucena-Agell D., Bucher P., Jansen R., Irschik H., Müller R., Paterson I., Díaz J.F., Altmann K.-H. (2018). A fluorescense anisotropy assay to discover and characterize ligands targeting the maytansine site of tubulin. Nat. Commun..

[15] Bold C.P., Lucena-Agnel D., Olive M.A., Diaz J.F., Altmann K. (2023). Synthesis and Biological Evaluation of C(13)/C(13’)-Bis(desmethyl)disorazole Z. Angew. Chem. Int. Ed..

