# Supplementary material for: Synthesis of a Non-Symmetrical Disorazole C1-Analogue and Its Biological Activity
Source: Molecules. 2024 Mar 1;29(5):1123. doi: 10.3390/molecules29051123 (PMC10934378; doi:10.3390/molecules29051123)

Table of Contents

^{1}H and ^{13}C NMR spectra 2

^{1}H and ^{13}C NMR spectra

^{1}H NMR (400 MHz, CD_3OD)

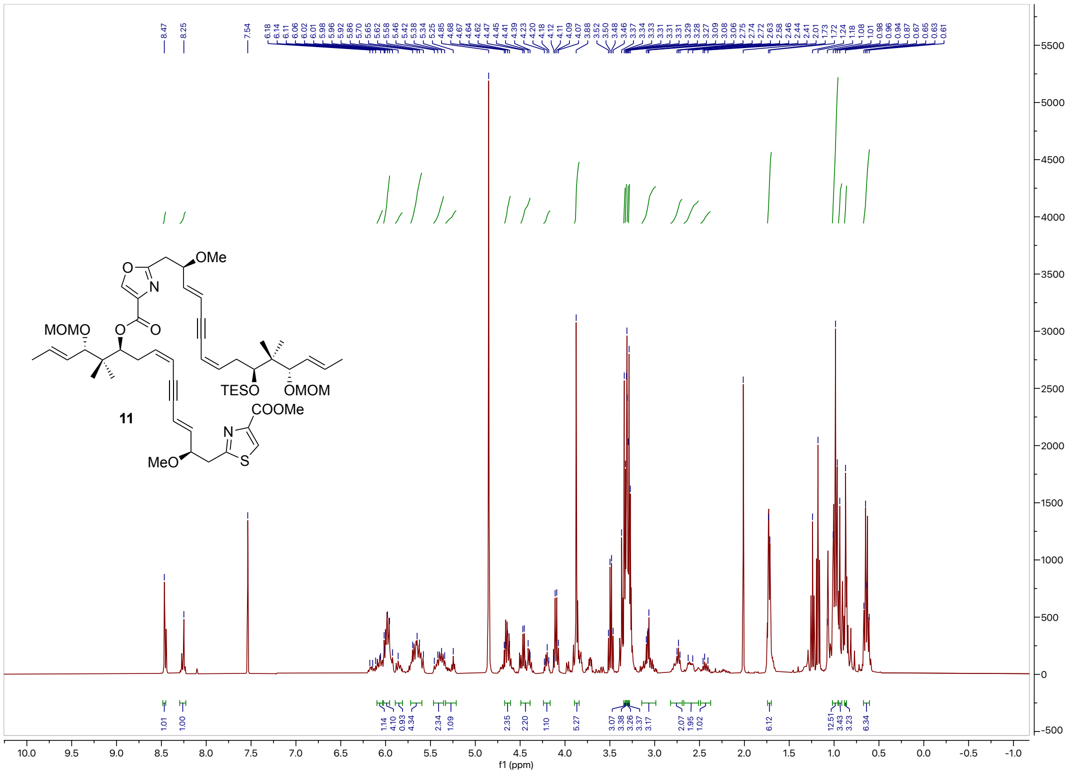

^{13}C NMR (101 MHz, CD_3OD)

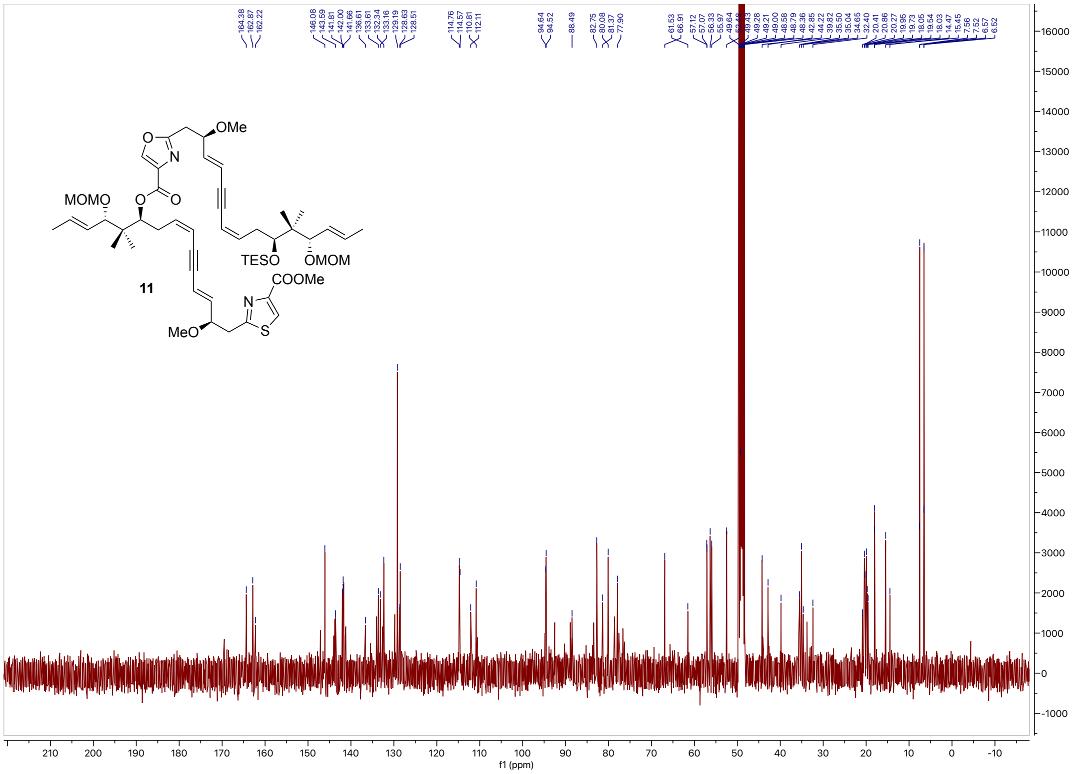

^{1}H NMR (400 MHz, $CDCl_3$)

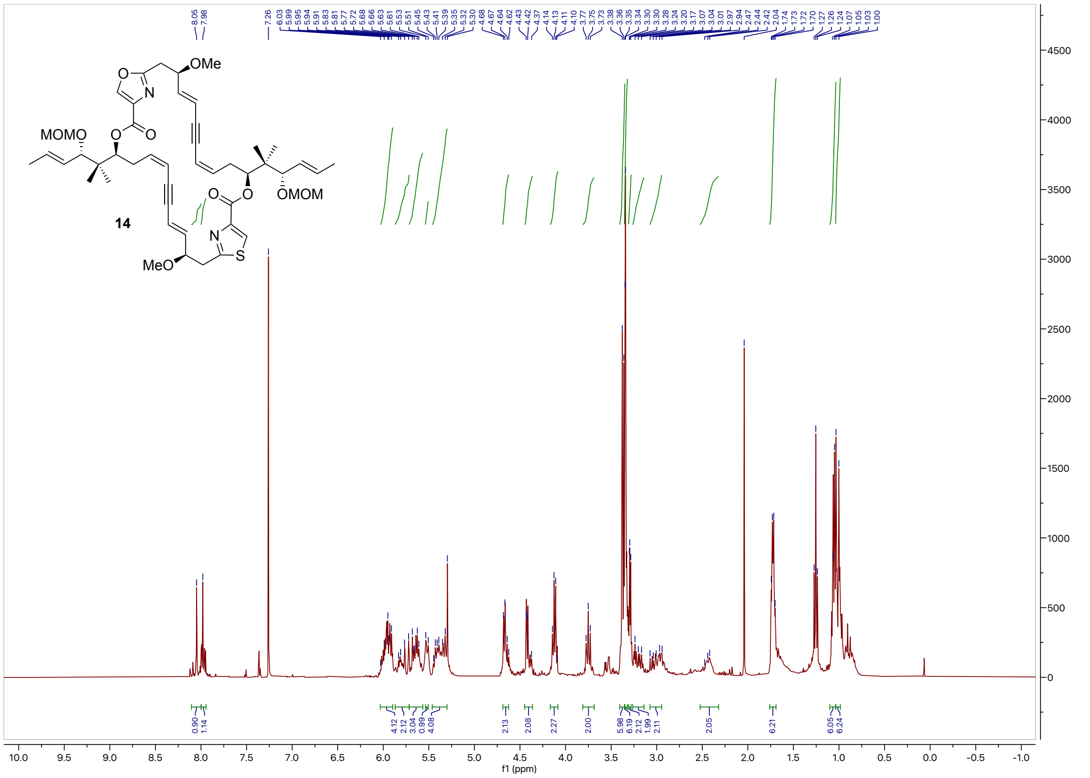

^{13}C NMR (101 MHz, $CDCl_3$)

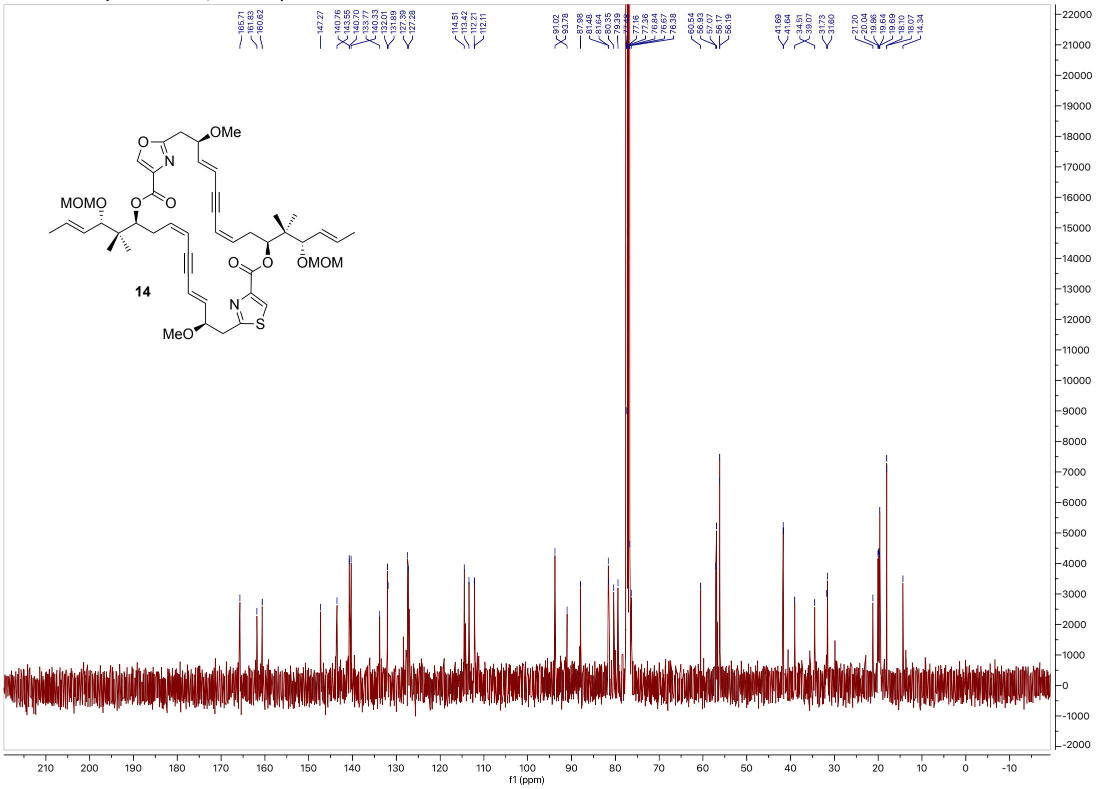

^{1}H NMR (600 MHz, $CDCl_3$)

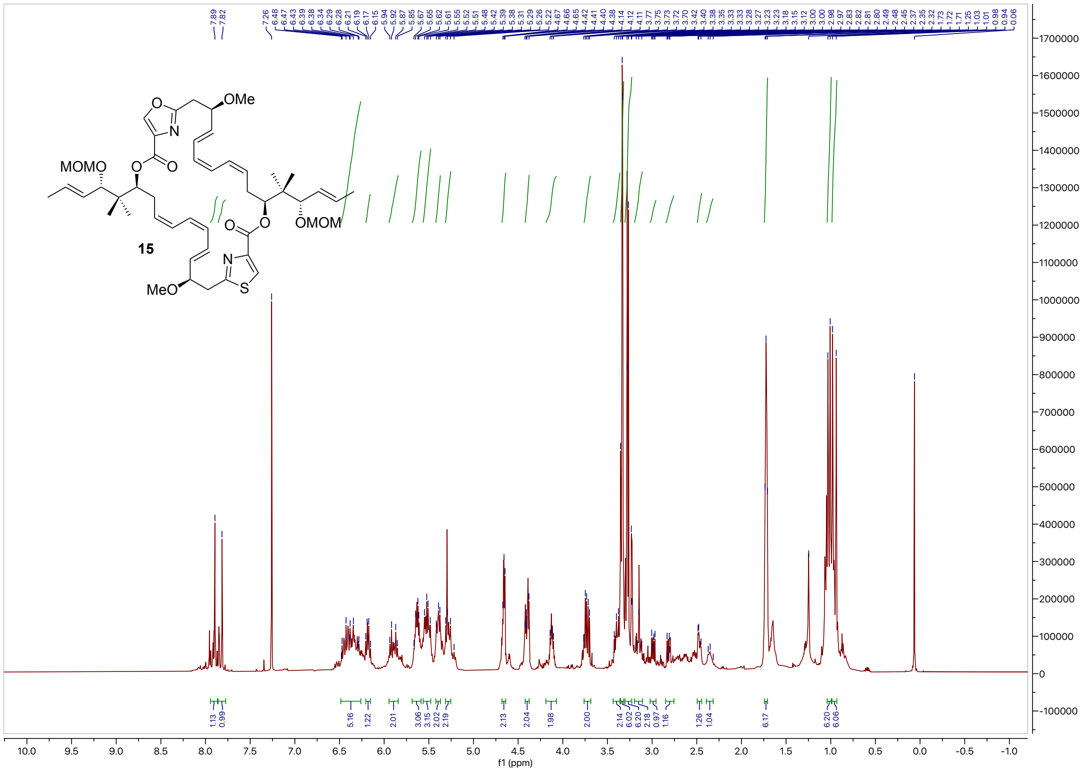

^{13}C NMR (151 MHz, $CDCl_3$)

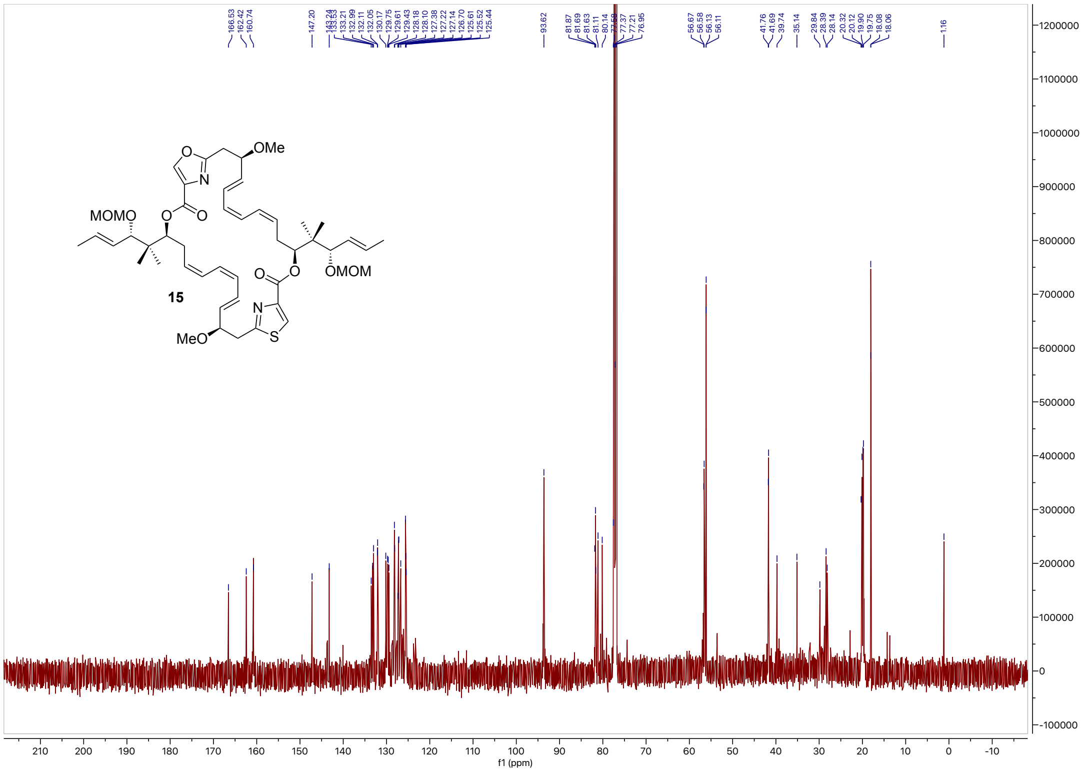

^{1}H NMR (600 MHz, CD_3OD)

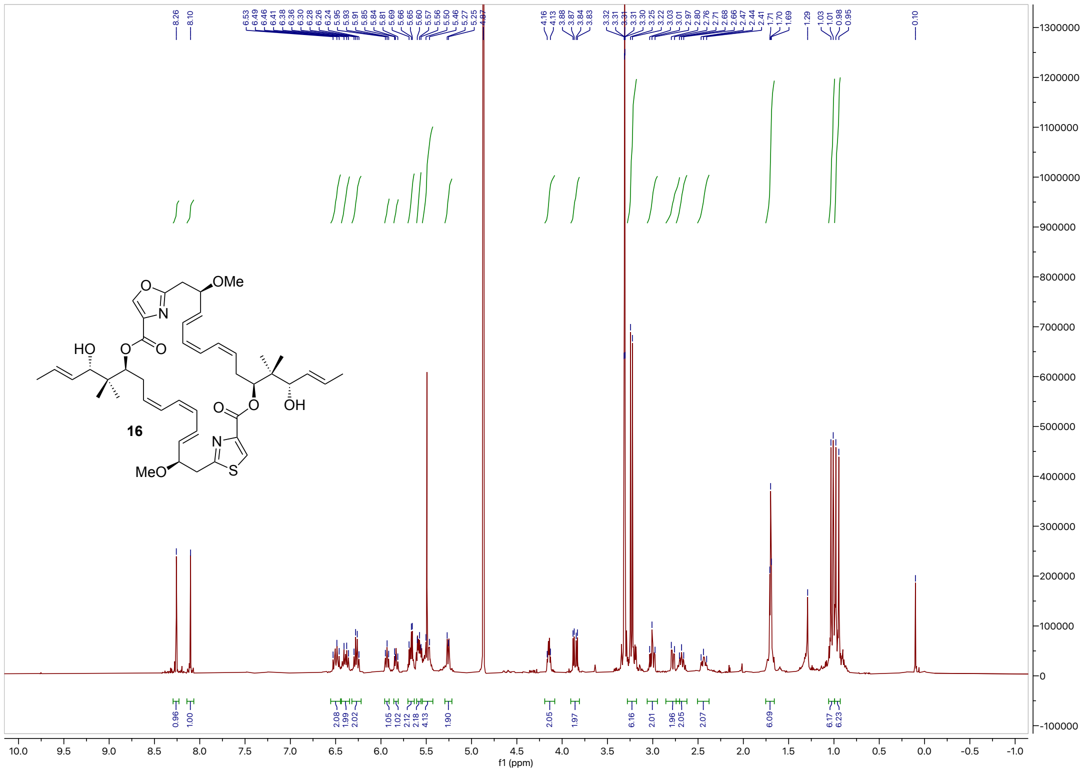

^{13}C NMR (151 MHz, CD_3OD)

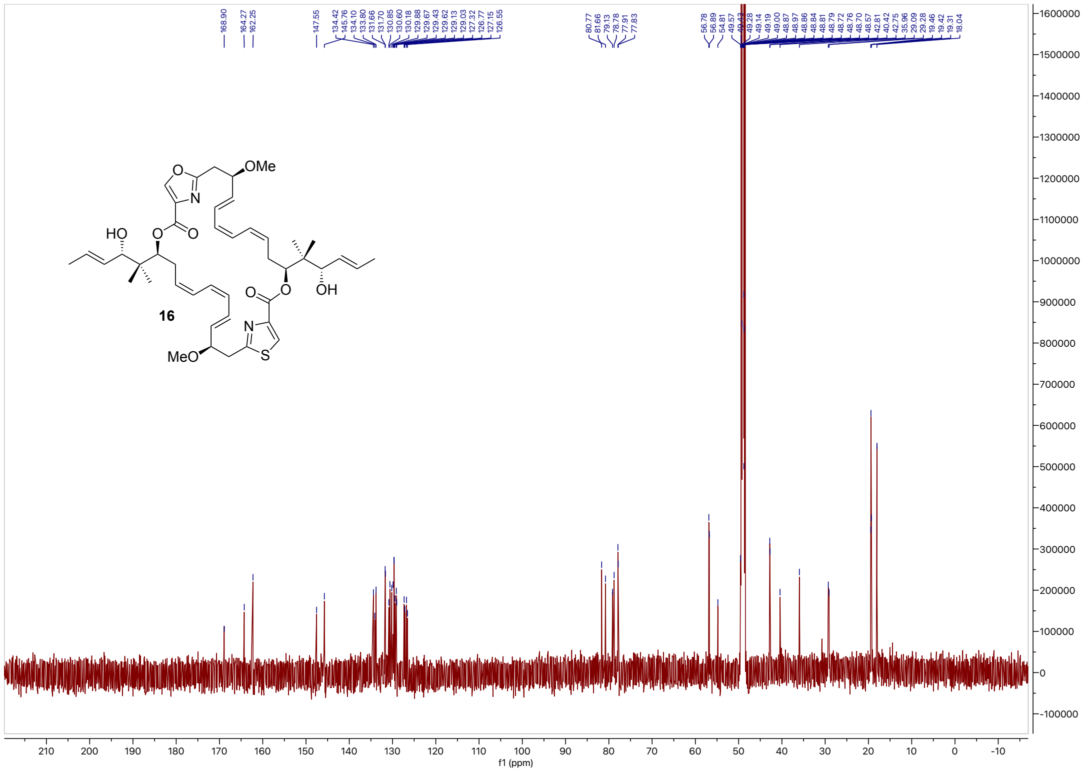

Supplement: Supplementary file 1 [file molecules-29-01123-s001.zip › molecules-2888882-supplementary.pdf]
